# Vfold: A Web Server for RNA Structure and Folding Thermodynamics Prediction

**DOI:** 10.1371/journal.pone.0107504

**Published:** 2014-09-12

**Authors:** Xiaojun Xu, Peinan Zhao, Shi-Jie Chen

**Affiliations:** Department of Physics and Department of Biochemistry, University of Missouri, Columbia, Missouri, United States of America; University of Georgia, United States of America

## Abstract

**Background:**

The ever increasing discovery of non-coding RNAs leads to unprecedented demand for the accurate modeling of RNA folding, including the predictions of two-dimensional (base pair) and three-dimensional all-atom structures and folding stabilities. Accurate modeling of RNA structure and stability has far-reaching impact on our understanding of RNA functions in human health and our ability to design RNA-based therapeutic strategies.

**Results:**

The Vfold server offers a web interface to predict (a) RNA two-dimensional structure from the nucleotide sequence, (b) three-dimensional structure from the two-dimensional structure and the sequence, and (c) folding thermodynamics (heat capacity melting curve) from the sequence. To predict the two-dimensional structure (base pairs), the server generates an ensemble of structures, including loop structures with the different intra-loop mismatches, and evaluates the free energies using the experimental parameters for the base stacks and the loop entropy parameters given by a coarse-grained RNA folding model (the Vfold model) for the loops. To predict the three-dimensional structure, the server assembles the motif scaffolds using structure templates extracted from the known PDB structures and refines the structure using all-atom energy minimization.

**Conclusions:**

The Vfold-based web server provides a user friendly tool for the prediction of RNA structure and stability. The web server and the source codes are freely accessible for public use at “http://rna.physics.missouri.edu”.

## Introduction

The increasing discoveries of noncoding RNAs demand more than ever the information about RNA structures [1]–[5]. However, laborious, time-consuming X-ray crystallographic or NMR spectroscopic measurements alone cannot catch up the pace with the rapidly increasing number of biologically significant RNAs such as noncoding regulatory RNAs. As a result, RNA structural genomics cannot just rely on the experimental determination of the structures. This underscores the request for accurate computational models of RNA structure prediction.

RNA structures can be described at the two-dimensional (2D) and three-dimensional (3D) levels, respectively. A 2D structure is defined by the base pairs contained in the structure. Helices and loops, as defined by the base pairs contained in the structure, can be diagrammatically depicted by an RNA 2D structure. The 2D structure of an RNA provides the structural constraints to the formation of the 3D structure [6]–[9], where helices and loops are assembled in the 3D space. RNA free energy landscape can have multiple free energy minima [10]–[13]. Therefore, an RNA can often adopt multiple stable and metastable structures.

Computational prediction for RNA 2D structures falls into two general categories [14]: sequence comparison [15]–[18] and free energy minimization [19]–[25]. Sequence comparison-based methods rely on base covariation and can usually only infer the information about the canonical base pairs. The inclusion of non-canonical base pairs can cause covariation analysis much more convoluted [26]. However, non-canonical base pairs such as mismatched base pairs in the loop regions, may be crucial for folding stability and 3D structure folding. For example, non-canonical base pairs can influence the loop and junction structures and thus play an critical role in determining helix orientations. The accuracy of computational prediction is usually better for methods that consider “fold recognition” [27]: structure is usually more conserved than sequence and the functional core regions are usually more conserved at all levels. Therefore, computational methods are highly useful and reliable for structures with known homologous folds or structures with sufficient auxiliary structural data. However, these methods depend on the availability of homologous sequences, which significantly limits their applicability.

Structure prediction algorithms based on free energy minimization search for the structure or suboptimal structures with the lowest free energy from an ensemble of possible structures. Most of the algorithms employ the same empirical thermodynamic parameters (the Turner parameters [28]) for the different secondary structural elements based on the nearest-neighbor model. However, unlike the entropy (free-energy) parameters for simple loops (hairpin, bulge, and internal loops), which have been determined from thermodynamic experiments [28], quantitative understanding of many other interactions remains very limited. Moreover, because of the possible conformational coupling between the loops, the loop entropies are not additive for tertiary motifs such as loop-loop kissing contacts [29], [30]. For such cases, thermodynamic experiments alone are not sufficient to directly provide loop entropies and free energies due to the complexity of the problem.

Current RNA folding algorithms for 3D structures are generally limited to simple (short) structures. Further development of the models is hampered by several challenges including conformational sampling and evaluation of the energies for the tertiary contacts. Combined with discrete molecular dynamics (DMD) [31], coarse-grained approaches [31]–[33] can be used to predict structures as well as folding mechanisms with knowledge-based potentials derived from known structures. Structure assembly approaches [26], [34]–[36], based on the assumption that 3D fold can be recognized by the alignment of sequences and secondary structure patterns, have shown promising results in RNA 3D structure predictions. However, one of the common limitations to the structure assembly approaches is the degree of divergence of the fragment library [37], [38].

The recently developed Vfold model is a statistical mechanics-based RNA folding model [36], [39]–[42] that can predict RNA 2D and 3D structures as well as RNA folding thermodynamic stabilities from RNA sequence. In this report, we briefly describe the underlying algorithm and the practical usage of a web server for the Vfold model (http://rna.physics.missouri.edu). The server provides predictions for the structure and melting thermodynamics for user-provided RNA sequences. The results from the server, in combination with experimental data, may offer useful insights into RNA structure and function.

## Methods

The Vfold model was first reported in 2005 for RNA secondary structure prediction [39]. Since then, the model has been extended to predict the structures and folding thermodynamics of H-type pseudoknots and RNA/RNA complexes [40]–[42]. Furthermore, Vfold was developed to predict 3D all-atom structures using a physics-based de novo method [36]. Below we describe several unique features of the Vfold model. The detailed underlining algorithms can be found in the published papers [36], [39]–[42] and in the Supporting Information (file Data S1) of this paper.

### Features of the Vfold algorithm

One of the unique features of the Vfold model for 2D structure (base pairs) prediction is its ability to compute the RNA motif-based loop entropies. Using the virtual bonds to represent the backbone conformations, the model samples fluctuations of loops/junction conformations in the 3D space through conformational enumeration [39] (see Figure S1 in Data S1 for details). By calculating the probability of loop formation, the model can give the conformational entropy parameters for the formation of the different types of loops such as pseudoknot loops. The model has the advantage of accounting for chain connectivity, exclude volume and the completeness of conformational ensemble. Studies by us and other groups show that an accurate entropy parameter improves the prediction of RNA secondary structures and thermodynamic stabilities [39]–[43].

Another notable feature of Vfold model is its ability to model intraloop mismatched base pairs for RNA loops (see Figure S2 in Data S1 for details). By enumerating all the possible (sequence-dependent) intra-loop mismatches, the Vfold model can partially account for the sequence-dependence of the loop free energy. Therefore, the Vfold-predicted loop free energy is not only loop size-dependent but also sequence-dependent. The model provides a unique tool for predicting many important information that cannot be obtained through traditional methods. For example, the model can calculate the dramatic decrease in loop entropy upon the formation of mismatched base pairs in a loop. The model can predict the populational distribution of the different loop conformations that contain the different intra-loop mismatches. The predicted mismatched base pairs provide constraints to otherwise flexible loop structures.

For a given 2D structure, the Vfold-based 3D structure prediction method [36] searches for the appropriate template for each loop/junction in the structure, and assembles the 3D template structures into a scaffold for further structure refinement. In comparison with other template-based (structure assembly) methods such as FARNA/FARFAR [34], [35] and MC-Sym [26], which sample structures from small fragments of the known RNA structures, the Vfold-based method uses motif-based instead of fragment-based templates. The main advantage of the multi-scale approach used in the Vfold 3D modeling [36] is that the virtual bond tertiary structure as the initial state may already lie in the free energy basin, so the structure refinement can avoid large structural rearrangements for the effective prediction of the final native structure.

### Energy parameters

The Vfold model provides pre-tabulated entropy parameters (available in the Vfold web server) for hairpin loops [39], internal/bulge loops [39], H-type pseudoknots with/without inter-helix junction [40], [41] and hairpin-hairpin kissing motifs [42]. For free energy-based RNA structure modeling, the predicted structures and thermodynamic stabilities could be sensitive to the choice of energy parameters. Therefore, the server provides predictions based on two different sets of the thermodynamic parameters for base stacks, including mismatched base stacks: (1) from the Turner parameters 04 version [28], and (2) from the MFOLD 2.3 version [20].

### 3D template library

**Figure 1:**
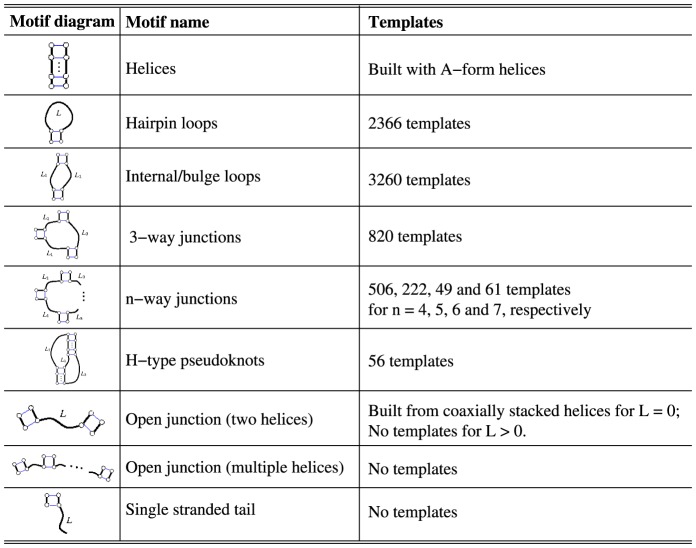
RNA 3D template structure database.

To construct the template library, Vfold classifies all the known structures into different motifs, such as helices, hairpin loops, internal/bulge loops, pseudoknots, N-way junctions (N
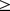
3) (see Figure.1). The motif-based template library was built from 2621 PDB structures, including all the PDB entries released before January of 2014. It includes RNA-involved complexes except RNA/DNA hybrids. The redundant templates for those with root mean square deviation (RMSD) 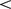
1.5 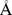 for the same motif, same size and identical sequence are removed. The complete list of the non-redundant 3D template list can be found in the Vfold web server.

## Results

The Vfold server contains three parts: (a) *Vfold2D* predicts the RNA 2D structure (pseudoknotted or non-pseudoknotted) from the sequence, (b) *VfoldThermal* predicts the melting curve (folding thermodynamics) from the sequence, and (c) *Vfold3D* predicts RNA 3D structure for a given 2D structure and the sequence. The computational time scales with the chain length *N* as *O*(*N* ^6^) and the memory scales as *O*(*N* ^2^) for Vfold2D and VfoldThermal. To avoid long computational time, the current version of the Vfold server restricts the RNA sequence length up to 140 nts.

### Vfold2D: Predicting RNA 2D structures from the sequence

**Figure 2:**
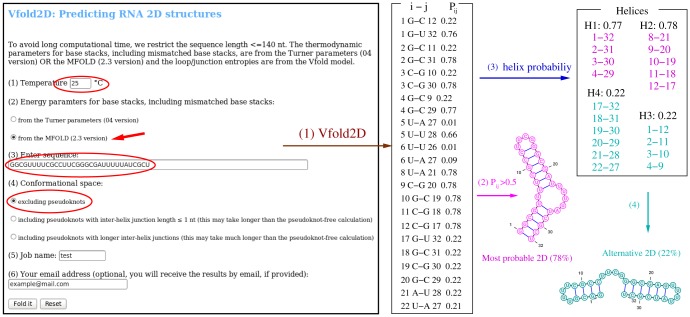
An example of Vfold2D prediction: the input information highlighted in the snapshot of the Vfold2D web server are the sequence (32 nts in this example), the temperature (25°C), the energy parameters used for base stacks (from MFOLD in this example) the structural type (non-pseudoknotted in this example). (1) Vfold2D gives a list of base pair probabilities P*_ij_* (in txt format) between nucleotides *i* and *j*. For example, the probability of forming G1-C12 base pair is 0.22. (2) The most probable 2D structure is derived from the base pairs with P*_ij_*>0.5. In this example, the predicted most probable 2D structure (plotted by VARNA in the figure) has the probability of 0.78. (3) Vfold2D also predicts all the possible helices from the predicted base pair probabilities. (4) Possible alternative structures can be found from the helix and base pair probabilities (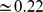 in this example).

The input of Vfold2D is the sequence in plain text form (see the snapshot of Vfold2D web server in Fig. 2). The default temperature for Vfold2D is 37°C. Users have the option to change the temperature to other values. Users have the option to use the base stacking energy parameters either from Turner's parameters or from the MFOLD. Users also have the option to choose the type of structures:

1. *Excluding pseudoknot*: Only non-pseudoknotted secondary structures are included in the structure prediction;
2. *Including pseudoknots with inter-helix junction length* 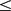 *1 nt*: All the possible non-pseudoknotted secondary structures and H-type pseudoknots with inter-helix junction of length 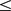
1 nt are considered in the calculation. It may take a much longer computational time than the pseudoknot-free calculations.
3. *Including pseudoknots with longer inter-helix junctions*: all the possible non-pseudoknotted secondary structures and H-type pseudoknots with inter-helix junction of any length are considered in the calculation. The computation may take much longer time than the calculation with pseudoknots of inter-helix junction length 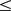
1 nt.

The Vfold2D server generates three files:

1. Base pair probabilities (in *txt* format).
2. Probabilities for the formation of the possible helices (including the native and alternative helices) (in *txt* format).
3. Predicted 2D structures (in *eps* format) plotted by VARNA [44].

We recommend users to consider the possible alternative structures from the base pair probabilities and helix probabilities (the first two output files above).

Fig. 2 shows an example of Vfold2D prediction for a 32-nt sequence [45]. With conformational sampling for the non-pseudoknotted structures, Vfold2D predicts the possible (including the alternative) helices from the base pair probabilities P*_ij_* based on the premise that base pairs (helices) in the same structure have the same level of probabilities of formation. The dominant 2D structure is identified from the base pairs of the largest probability. Fig. 2 shows an RNA that has two sets of helices. One set shown in magenta has the probability of 0.78. This is the most probable structure. Another set of helices in cyan with probability 0.22 gives an alternative structure. The predicted bistable structures agree with the NMR results [45].

### VfoldThermal: predicting RNA melting curves

**Figure 3:**
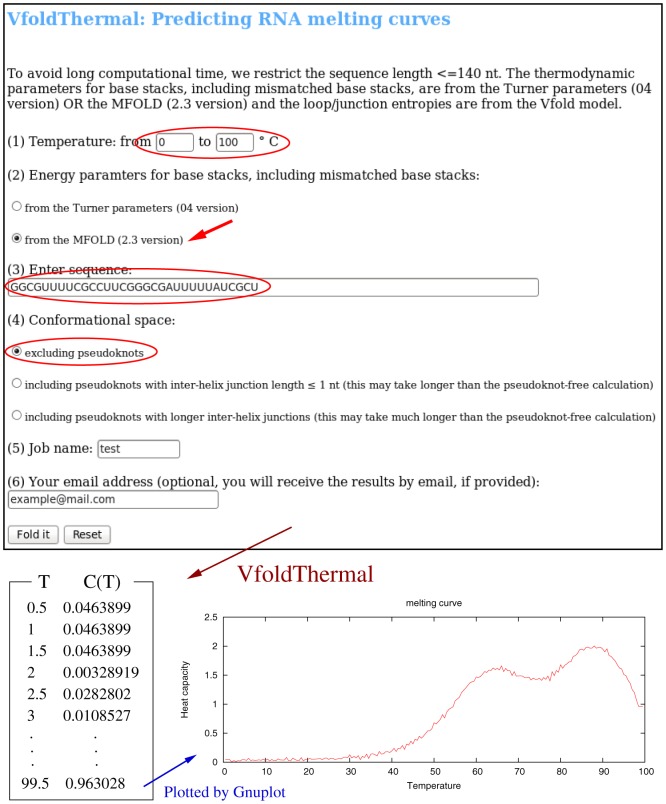
An example of the VfoldThermal prediction: the inputs highlighted in the snapshot of VfoldThermal web server are the sequence (32 nts in this example) with the temperature range of 0°C–100°C, the energy parameters used for base stacks (from MFOLD in this example) and the structure type (non-pseudoknotted in this example). From the temperature dependence of the partition function Q(*T*), VfoldThermal gives a list of temperature-dependent heat capacity C(*T*), with temperature interval of 0.5°C. The *eps* format of melting curve is generated by Gnuplot.

VfoldThermal predicts the heat capacity *C*(*T*) melting curves from the temperature-dependence of the partition function Q(*T*) for the conformational ensemble chosen by the user. The server provides the results in text format as well as in eps format plotted by Gnuplot. The input of VfoldThermal is the same as those for the Vfold2D, except for the temperature range in VfoldThermal (see the snapshot of VfoldThermal web server in Fig. 3).

For the example shown in Fig. 3, with the same input as for Vfold2D in Fig. 2, VfoldThermal calculates the partition function Q(*T*) for all the non-pseudoknotted structures for temperature range 0°C–100°C with the temperature step of 0.5°C. The predicted heat capacity (melting curve) shows two peaks around 60 and 90°C, respectively. The peaks correspond to the melting of the two helices in the predicted structures in Fig. 2, respectively.

### Vfold3D: Predicting RNA 3D structure

**Figure 4:**
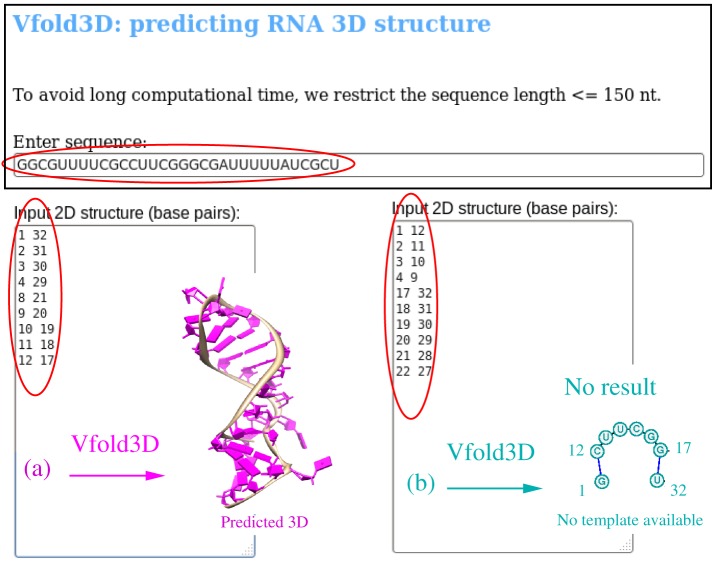
An example of the Vfold3D prediction: the snapshot of Vfold3D web server highlights the input sequence (32 nts for this example) and the 2D structures as defined by the base pairs. (a) For the most probable 2D structure shown in Fig. 2, Vfold3D predicts 3D structure based on the templates from the known structures. (b) For the predicted alternative structure shown in Fig. 2, Vfold3D cannot predict the 3D structure due to the lack of the available template for the single-stranded chain between the helices.

The input data of Vfold3D are the RNA sequence and the 2D structure (base pairs) (see the snapshot of the Vfold3D web server in Fig. 4). The output of Vfold3D is a PDB file for the predicted all-atom 3D structure(s). Because the current version of Vfold3D is template-based, no 3D structure will be predicted if a proper template cannot be found.

Currently, due to the limited structural template database, the current version of Vfold3D can only predict the 3D structures with hairpin loops, internal/bulge loops, N-way (2<N<8) junctions and pseudoknots. For example, as listed in Figure.1, there is no templates available for the open motifs (single strand tails and tandem helices except for coaxially stacked helices). Therefore, it is recommended to remove the single strand tails before submitting jobs to Vfold3D. With the increasing number of the known RNA structures, the larger and more divergent pools of the known loop/junction structures with the different types and different sizes would lead to better predictions from the Vfold3D.

For the RNA in Fig. 2, Vfold2D predicts two alternative 2D structures. As shown in Fig. 4, for the most probable 2D structure, Vfold3D predicts one 3D structure. For the alternative 2D structure, which consists of two hairpins connected by a single-strand loop, Vfold3D yields no 3D structure because of the lack of the templates for the UUCG single-stranded open junction between the two hairpins.

### Vfold output

Once a calculation is submitted, a notification page containing the job information (job name, e-mail address (optional) and the job status) is displayed. When the calculation is completed, the Vfold web server sends out an e-mail (if provided) notification with the predicted results attached. It is recommended to bookmark the job-specific notification page for later check of the job status and for downloading Vfold predicted results, since *Vfold2D* and *VfoldThermal* might take a long computational time (hours or even longer) depending on the sequence length. An online *README* file about the interpretation of the Vfold predictions is available on the Vfold web server.

## Conclusion

The Vfold package is developed to predict RNA structures and folding thermodynamics. The web server will be updated continuously with the development of new Vfold-based algorithms for RNA folding. In the future development, we plan to add structure predictions for the formation of RNA-RNA complexes. We will also add the effect of the ion-dependent electrostatic free energies and the heat capacity effect, which can cause the temperature-dependence of the enthalpy and entropy parameters for the loop and base stack formations, to the melting curve calculations and structure predictions.

## Supporting Information

Data S1 (PDF)

## References

[1] Doudna JA, Cech TR (2002) The chemical repertoire of natural ribozymes. Nature 418: 222–228. 12110898 10.1038/418222a

[2] Bachellerie JP, Cavaille J, Huttenhofer A (2002) The expanding snoRNA world. Biochimie 84: 774–790. 10.1016/s0300-9084(02)01402-5 12457565

[3] Gong C, Maquat LE (2011) lncRNAs transactivate STAU1-mediated mRNA decay by duplexing with 3′ UTRs via Alu elements. Nature 470: 284–288. 21307942 10.1038/nature09701 PMC3073508

[4] Bartel DP (2009) MicroRNAs: target recognition and regulatory functions. Cell 136: 215–233. 19167326 10.1016/j.cell.2009.01.002 PMC3794896

[5] Kertesz M, Iovino N, Unnerstall U, Gaul U, Segal E (2007) The role of site accessibility in microRNA target recognition. Nat. Genet 39: 1278–1284. 17893677 10.1038/ng2135

[6] Tinoco I, Bustamante C (1999) How RNA folds. J. Mol. Biol. 293: 271–281. 10550208 10.1006/jmbi.1999.3001

[7] Onoa B, Tinoco I (2004) RNA folding and unfolding. Curr. Opin. Struct. Biol. 14: 374–379. 10.1016/j.sbi.2004.04.001 15193319

[8] Hajdin CE, Ding F, Dokholyan NV, Weeks KM (2010) On the significance of an RNA tertiary structure prediction. RNA 16: 1340–1349. 20498460 10.1261/rna.1837410 PMC2885683

[9] Xia Z, Bell DR, Shi Y, Ren P (2013) RNA 3D structure prediction by using a coarse-grained model and experimental data. J Phys Chem B 117: 3135–3144. 23438338 10.1021/jp400751w

[10] Xu X, Chen S-J (2012) Kinetic mechanism of conformational switch between bistable RNA hairpins. J Am Chem Soc 134: 12499–12507. 22765263 10.1021/ja3013819 PMC3427750

[11] Bussi G, Gervasio FL, Laio A, Parrinello M (2006) Free-energy landscape for beta hairpin folding from combined parallel tempering and metadynamics. J Am Chem Soc 128: 13435–13441. 17031956 10.1021/ja062463w

[12] Senter E, Dotu I, Clote P (2014) Efficiently computing the 2D energy landscape of RNA. J Math Biol In Press. 10.1007/s00285-014-0760-4 24515409

[13] Lin JC, Thirumalai D (2008) Relative stability of helices determines the folding landscape of adenine riboswitch aptamers. J Am Chem Soc 130: 14080–14081. 18828635 10.1021/ja8063638

[14] Shapiro BA, Yingling YG, Kasprzak W, Bindewald E (2007) Bridging the gap in RNA structure prediction. Curr. Opin. Struct. Biol. 17: 157–165. 17383172 10.1016/j.sbi.2007.03.001

[15] Havgaard JH, Lyngso RB, Gorodkin J (2005) The FOLDALIGN web server for pairwise structural RNA alignment and mutual motif search. Nucleic Acids Res 33: W650–W653. 15980555 10.1093/nar/gki473 PMC1160234

[16] Bernhart SH, Hofacker IL, Will S, Gruber AR, Stadler PF (2008) RNAalifold: improved consensus structure prediction for RNA alignments. BMC Bioinformatics 9: 474. 19014431 10.1186/1471-2105-9-474 PMC2621365

[17] Sato K, Hamada M, Asai K, Mituyama T (2009) CENTROIDFOLD: a web server for RNA secondary structure prediction. Nucleic Acids Res 37: W277–W280. 19435882 10.1093/nar/gkp367 PMC2703931

[18] Mathews DH, Turner DH (2002) Dynalign: an algorithm for finding the secondary structure common to two RNA sequences. J Mol Biol 317: 191–203. 11902836 10.1006/jmbi.2001.5351

[19] Mathews DH, Turner DH (2006) Prediction of RNA secondary structure by free energy minimization. Curr. Opin. Struct. Biol. 16: 270–278. 16713706 10.1016/j.sbi.2006.05.010

[20] Zuker M (2003) Mfold web server for nucleic acid folding and hybridization prediction. Nucleic Acids Res 31: 3406–3415. 12824337 10.1093/nar/gkg595 PMC169194

[21] Hofacker IL (2003) Vienna RNA secondary structure server. Nucleic Acids Res. 31: 3429–3431. 12824340 10.1093/nar/gkg599 PMC169005

[22] Bellaousov S, Reuter JS, Seetin MG, Methews DH (2013) RNAstructure: web servers for RNA secondary structure prediction and analysis. Nucleic Acids Res 41: W471–W474. 23620284 10.1093/nar/gkt290 PMC3692136

[23] Xayaphoummine A, Bucher T, Isambert H (2005) Kinefold web server for RNA/DNA folding path and structure prediction including pseudoknots and knots. Nucleic Acids Res 33: W605–W610. 15980546 10.1093/nar/gki447 PMC1160208

[24] Ren J, Rastegari B, Condon A, Hoos HH (2005) HotKnots: Heuristic prediction of RNA secondary structures including pseudoknots. RNA 11: 1494–1504. 16199760 10.1261/rna.7284905 PMC1370833

[25] Hajiaghayi M, Condon A, Hoos HH (2012) Analysis of energy-based algorithms for RNA secondary structure prediction BMC Bioinformatics, 13, 22. 10.1186/1471-2105-13-22 PMC3347993 22296803

[26] Parisien M, Major F (2008) The MC-fold and MC-sym pipeline infers RNA structure from sequence data. Nature 452: 51–55. 18322526 10.1038/nature06684

[27] Rother K, Rother M, Boniecki M, Puton T, Bujnicki JM (2011) RNA and protein 3D structure modeling: similarities and differences. J. Mol. Model 17: 2325–2336. 10.1007/s00894-010-0951-x PMC3168752 21258831

[28] Turner DH, Mathews DH (2010) NNDB: the nearest neighbor parameter database for predicting stability of nucleic acid secondary structure. Nucleic Acid Res 38: D280–D282. 19880381 10.1093/nar/gkp892 PMC2808915

[29] Cao S, Xu X, Chen S-J (2014) Predicting structure and stability for RNA complexes with intermolecular loop-loop base-pairing. RNA 20: 835–845. 24751648 10.1261/rna.043976.113 PMC4024638

[30] Zhang J, Lin M, Chen R, Wang W, Liang J (2008) Discrete state model and accurate estimation of loop entropy of RNA secondary structures. J Chem Phys 128: 125107. 18376982 10.1063/1.2895050 PMC2494904

[31] Ding F, Sharma S, Chalasani P, Demidov VV, Broude NE, et al (2008) Ab initio RNA folding by discrete molecular dynamics: from structure prediction to folding mechanisms. RNA 14: 1164–1173. 18456842 10.1261/rna.894608 PMC2390798

[32] Sharma S, Ding F, Dokholyan NV (2008) iFoldRNA: three-dimensional RNA structure prediction and folding. Bioinformatics 24: 1951–1952. 18579566 10.1093/bioinformatics/btn328 PMC2559968

[33] Xia Z, Gardner DP, Gutell RR, Ren P (2010) Coarse-grained model for simulation of RNA three-dimensional structures. J Phys Chem B 114: 13497–13506. 20883011 10.1021/jp104926t PMC2989335

[34] Das R, Baker D (2007) Automated de novo prediction of native-like RNA tertiary structures. Proc Natl Acad Sci USA 104: 14664–14669. 17726102 10.1073/pnas.0703836104 PMC1955458

[35] Das R, Karanicolas J, Baker D (2010) Atomic accuracy in predicting and designing noncanonical RNA structure. Nat Methods 7: 291–294. 20190761 10.1038/nmeth.1433 PMC2854559

[36] Cao S, Chen S-J (2011) Physics-based de novo prediction of RNA 3D structures. J. Phys. Chem. B 115: 4216–4226. 21413701 10.1021/jp112059y PMC3072456

[37] Petrov AI, Zirbel CL, Leontis NB (2011) WebFR3D-a server for finding, aligning and analyzing recurrent RNA 3D motifs. Nucleic Acids Res, 39, W50–W55 10.1093/nar/gkr249 PMC3125732 21515634

[38] Popenda M, Blazewicz M, Szachniuk M, Adamiak RW (2008) RNA FRABASE version 1.0: an engine with a database to search for the three-dimensional fragments within RNA structures. Nucleic Acids Res 36: D386–D391. 17921499 10.1093/nar/gkm786 PMC2238875

[39] Cao S, Chen S-J (2005) Predicting RNA folding thermodynamics with a reduced chain representation model. RNA 11: 1884–1897. 16251382 10.1261/rna.2109105 PMC1370876

[40] Cao S, Chen S-J (2006) Predicting RNA psuedoknot folding thermodynamics. Nucleic Acids Res. 34: 2634–2652. 16709732 10.1093/nar/gkl346 PMC1463895

[41] Cao S, Chen S-J (2009) Predicting structures and stabilities for H-type pseudoknots with inter-helix loop. RNA 15: 696–706. 19237463 10.1261/rna.1429009 PMC2661829

[42] Cao S, Chen S-J (2011) Structure and stability of RNA/RNA kissing complex: with application to HIV dimerization initiation signal. RNA 17: 2130–2143. 22028361 10.1261/rna.026658.111 PMC3222126

[43] Andronescu MS, Pop C, Condon AE (2010) Improved free energy parameters for RNA pseudoknotted secondary structure prediction. RNA 16: 26–42. 19933322 10.1261/rna.1689910 PMC2802035

[44] Darty K, Denise A, Ponty Y (2009) VARNA: Interactive drawing and editing of the RNA secondary structure Bioinformatics. 25: 1974–1975. 10.1093/bioinformatics/btp250 PMC2712331 19398448

[45] Hobartner C, Micura R (2003) Bistable secondary structures of small RNAs and their structural probing by comparative imino proton NMR spectroscopy. J Mol Biol 325: 421–431. 12498793 10.1016/s0022-2836(02)01243-3

