# Supplementary material for: Vfold: A Web Server for RNA Structure and Folding Thermodynamics Prediction
Source: PLoS One. 2014 Sep 12;9(9):e107504. doi: 10.1371/journal.pone.0107504 (PMC4162592; doi:10.1371/journal.pone.0107504)
Supplement: Data S1 — (PDF) [file pone.0107504.s001.pdf]

Supplementary Information:

Vfold: a web server for RNA structure and folding thermodynamics prediction

Xiaojun XU, Peinan ZHAO, and Shi-Jie CHEN
Department of Physics and Department of Biochemistry
University of Missouri, Columbia, MO 65211

Virtual bond representation and loop entropy calculation

Due to the rotameric nature of RNA backbone[1, 2], the backbone conformation of a nucleotide can be described by two virtual bonds instead of the original six-bond while keeping the realism of the conformational degrees of freedom (see Fig.S1a & b). The reduced conformational complexity enables conformational sampling and conformational entropy calculation through exact enumeration of the discrete conformations for the different structure types.

Currently, the Vfold model enumerates backbone conformations on a diamond lattice with three equiprobable torsional angles to sample fluctuations of loops/junctions in 3D space. By calculating the probability of loop formation, the model can give the conformational entropy parameters for the formation of the different types of loops such as pseudoknot loops. Fig.S1c shows an illustrative example for the entropy calculation for a hairpin loop. An advantage of the Vfold entropy calculation is its ability to account for chain connectivity, excluded volume effect and completeness of the conformational ensemble.

The Vfold model provides pre-tabulated entropy parameters for hairpin loops[3], internal/bulge loops[3], H-type pseudoknots with/without inter-helix junction[4, 5] and hairpin-hairpin kissing motifs[6].

Conformational ensemble and partition function

At the center of the prediction of the thermodynamic properties is the partition function. The partition function is the sum of the Boltzmann weight over all the possible structures. For a given RNA sequence, Vfold enumerates all the possible two-dimensional (2D) structures, including pseudoknots and secondary structures, using a recursive algorithm[3, 4, 5, 6]. For each (2D) structure, the free energies for canonical and non-canonical (mismatched) base stacks are calculated from Turner's experimental data [7] and the loop entropies are from the Vfold pre-tabulated parameters (see Fig. S1).

By calculating the total partition function $Q_{tot}(T)$ over the complete ensemble of RNA (2D) structures and the conditional partition function $Q_{ij}(T)$ for all the conformations that contain base pair (i, j) between nucleotide i and nucleotide j, Vfold computes the base-pairing probability as $P_{ij}(T)$=$Q_{ij}(T)$/$Q_{tot}(T)$. From $P_{ij}(T)$ for all the possible (i, j)'s, Vfold predicts all the stable structures, including the global minimum free energy structure. Moreover, from the temperature-dependent partition function $Q_{tot}(T)$, one can predict the melting curves and folding thermodynamics (equilibrium folding pathways) from the sequence.

Compared with the other free energy-based RNA 2D structure prediction models, like Mfold[8], RNAstructure[9], the Vfold model computes entropy parameters from explicit conformational sampling. Because the Vfold model gives the entropy for a given structure such as a loop structure with given intra-loop contacts, it allows us to enumerate all the possible intra-loop mismatches and compute the free energy for each given set of intra-loop mismatches. Summing over all the loop conformations gives the partition function, from which we predict the loop free energy; see Fig. S2 for illustration, We note that the mismatched base pairs (stacks) in a loop partially account for the intra-loop non-canonical interactions. Moreover, because the mismatch parameters are dependent on the base identity [7], the Vfold-predicted loop free energy is not only loop size-dependent but also sequence-dependent. Furthermore, in the Vfold model, the enthalpic contribution from the base pair mismatches makes the loop free energy not purely entropic.

The detailed algorithm for the enumeration of all the possible 2D structures including non-pseudoknotted secondary structures and H-type pseudoknots can be found in the published papers[3, 4, 5]. In summary, the non-

pseudoknotted structures are enumerated through a recursive algorithm and the pseudoknotted structures are generated through explicit enumeration of the loop and stem locations in the sequence and allowing the formation of non-pseudoknotted structures inside the loops.

Template-based 3D structure prediction

A 2D structure can correspond to a large number of three-dimensional (3D) structures due to the multiplicity of loop conformations. The Vfold model predict the 3D structure for a 2D structure based on the structural templates[10]. Vfold predicts RNA 3D structures by assembling 3D motifs. This approach is based on the structure of the whole motif[11, 12]. The method is differed from other structure assembly methods (such as FARNA/FARFAR[13, 14] and MC-Sym[15]), which sample structures from small fragments of known RNA structures.

Based on the 2D structure, Vfold first builds the 3D virtual bond structure. Helices are modeled as A-form virtual-bond helix structures. The loop/junction structures are built from the virtual bond conformations of the template structures. To identify the optimal template structure for the given loops/junctions, the model screens the template library according to the loop size (first) and the sequence (second) matches. If necessary, this step may involve sequence replacement in order to match the sequences in the template library. That's why we focus on the (virtual bond) backbone structure (without the bases) as the first step. With the template structure for the loops/junctions, the model then assembles the helix and loop 3D structures to construct the 3D scaffold of the whole RNA; See Fig. S3.

In the next step, based on the virtual bond scaffold, Vfold builds the all-atom RNA 3D structures (shown in Fig.S3) by adding bases to the virtual bond backbone according to the templates for base configurations. The final all-atom structure is refined by AMBER energy minimization[16]. For most predicted structures, we found that the minimization causes only small changes in the root mean square deviation (RMSD) of the structure. The main advantage of the multi-scale approach used in the Vfold modeling is that the virtual bond tertiary structures as the initial state may already lie in the free energy basin, so the structure refinement can avoid large structural rearrangements and can thus lead to the native structure effectively.

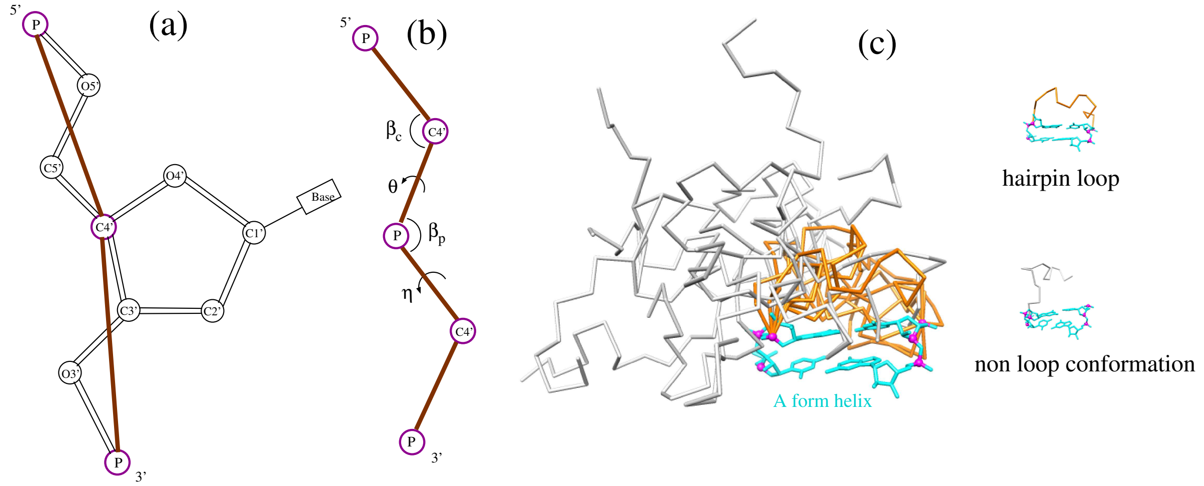

Figure S1: Virtual bond-based RNA folding model (Vfold): (a) Virtual bond model for RNA conformation. The original six-bond (on backbone) nucleotide is reduced to a two-bond unit. (b) The bond angles (β_c, β_p) and the torsional angles (θ, η) for the virtual bonds. RNA backbone conformations can be configured on a diamond lattice with bond length of $3.9\mathring{A}$, bond angle of $109.5°$ and three equiprobable torsional angles (60°, 180°, 300°). (c) Ensemble of conformations for the entropy calculation for a 6-nt RNA hairpin loop. Vfold enumerates exhaustively all the possible (virtual bonded) backbone conformations, and counts the numbers of viable hairpin loop Ω_{hairpin} and total Ω_{total} conformations, respectively. The entropy is estimated as $\Delta S = k_B \ln(\Omega_{\text{hairpin}}/\Omega_{\text{total}})$.

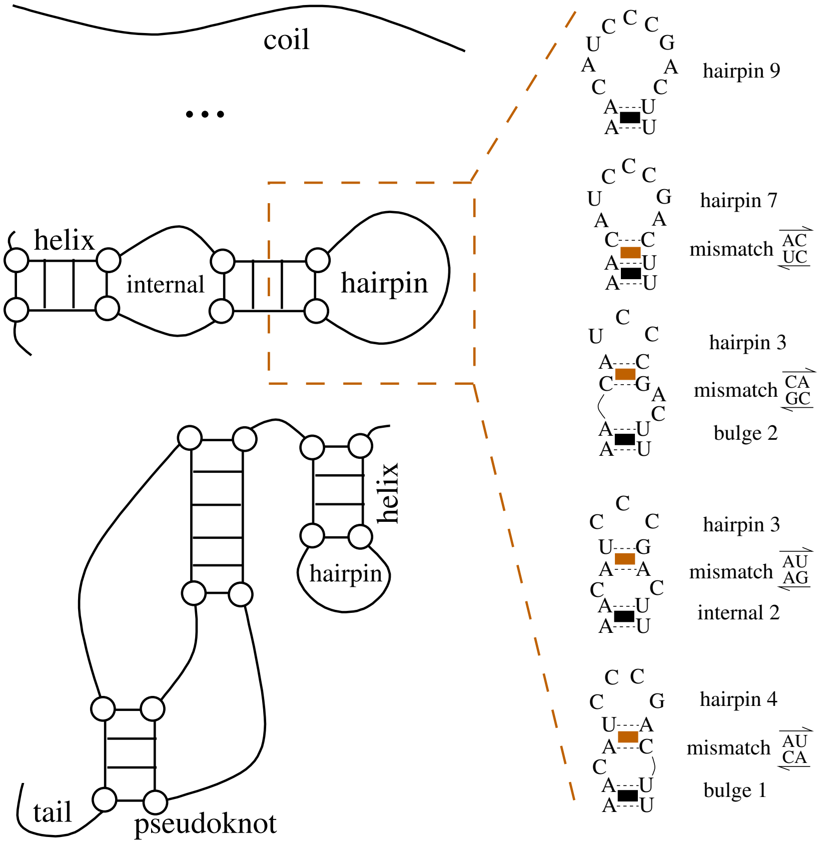

Figure S2: Ensemble of RNA structures: Vfold enumerates all the possible structures (left panel) including pseudoknots and secondary structures for a given RNA sequence. For each structure, the loop free energies are obtained by calculating the partition function over all the possible arrangements of the intra-loop mismatched base stacks. Shown in right panel is an example for the partition function calculation for a 9-nt hairpin loop closed by an A-U base pair. For this example, the ensemble of the loop conformations contains 5 different arrangements of mismatched base stacks within the loop.

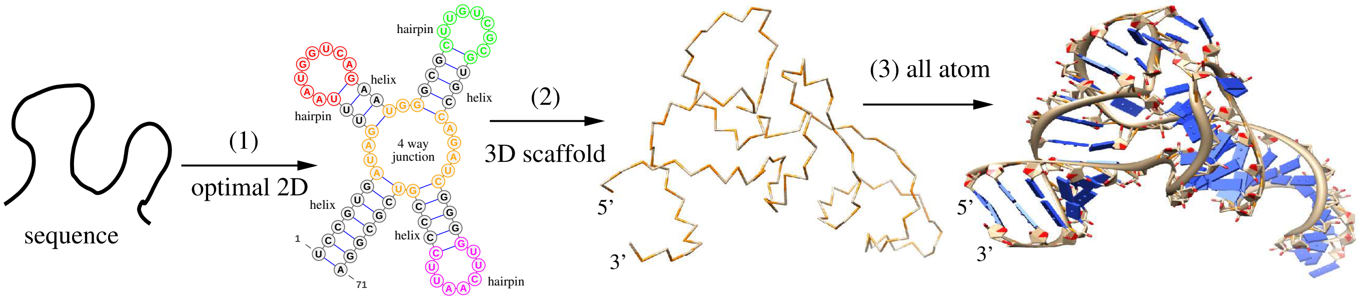

Figure S3: Multi-scaling strategy of Vfold 3D structure prediction: 3D scaffold is built based on the predicted 2D structure. Helices are modeled as A-form (virtual-bond) RNA helices. The optimal loop/junction virtual-bonded structures are selected from fragments of known structures. Then the atomistic 3D structures are built by adding bases to the virtual bond backbone according to the templates for base configurations. The final all-atom structure is refined by AMBER energy minimization to remove possible atomic clashes.

References

[1] Olson WK. (1980). Configurational statistics of polynucleotide chains. An updated virtual bond model to treat effects of base stacking. *Macromolecules*, **13**: 721-728.

[2] Duarte CM, Pyle AM. (1998). Stepping through an RNA structure: a novel approach to conformational analysis. *J Mol Biol*, **284**: 1465-1478.

[3] Cao S ,Chen S-J. (2005). Predicting RNA folding thermodynamics with a reduced chain representation model. *RNA*, **11**: 1884-1897.

[4] Cao S, Chen S-J. (2006). Predicting RNA psuedoknot folding thermodynamics. *Nucleic Acids Res.*, **34**: 2634-2652.

[5] Cao S, Chen S-J. (2009). Predicting structures and stabilities for H-type pseudoknots with inter-helix loop. *RNA*, **15**: 696-706.

[6] Cao S, Chen S-J. (2011). Structure and stability of RNA/RNA kissing complex: with application to HIV dimerization initiation signal. *RNA*, **17**: 2130-2143.

[7] Turner DH, Mathews DH. (2010). NNDB: the nearest neighbor parameter database for predicting stability of nucleic acid secondary structure. *Nucleic Acid Res.*, **38**: D280-D282.

[8] Zuker M. (2003). Mfold web server for nucleic acid folding and hybridization prediction. *Nucleic Acids Res.*, **31**: 3406-3415.

[9] Bellaousov S, Reuter JS, Seetin MG, Methews DH. (2013). RNAstructure: web servers for RNA secondary structure prediction and analysis. *Nucleic Acids Res*, **41**: W471-W474.

[10] Cao S, Chen S-J. (2011). Physics-based de novo prediction of RNA 3D structures. *J. Phys. Chem. B*, **115**: 4216-4226.

[11] Petrov AI, Zirbel CL, Leontis NB. (2011). WebFR3D-a server for finding, aligning and analyzing recurrent RNA 3D motifs. *Nucleic Acids Res*, **39**, W50-W55.

[12] Popenda M, Blazewicz M, Szachniuk M, Adamiak RW. (2008). RNA FRABASE version 1.0: an engine with a database to search for the three-dimensional fragments within RNA structures. *Nucleic Acids Res*, **36**, D386-D391.

[13] Das R, Baker D. (2007). Automated de novo prediction of native-like RNA tertiary structures. *Proc Natl Acad Sci USA*, **104**, 14664-14669.

[14] Das R, Karanicolas J, Baker D. (2010). Atomic accuracy in predicting and designing noncanonical RNA structure. *Nat Methods*, **7**, 291-294.

[15] Parisien M, Major F. (2008). The MC-fold and MC-sym pipeline infers RNA structure from sequence data. *Nature*, **452**: 51-55.

[16] Case DA, Babin V, Berryman JT, Betz RM, et al. (2014). AMBER 14. *University of California, San Francisco*.
